# Accuracy of Computer-Guided Implantology with Pilot Drill Surgical Guide: Retrospective 3D Radiologic Investigation in Partially Edentulous Patients

**DOI:** 10.3390/medicina59040738

**Published:** 2023-04-10

**Authors:** Federico Gelpi, Nicolò Modena, Alessandro Poscolere, Fabio Bernardello, Lorena Torroni, Daniele De Santis

**Affiliations:** 1 Head and Neck Department, Department of Surgery, Dentistry, Pediatrics and Gynecology, University of Verona, 37124 Verona, Italy; 2 Private Practitioner, 37124 Verona, Italy; 3 Unit of Epidemiology and Medical Statistics, Department of Diagnostics & Public Health, University of Verona, 37129 Verona, Italy

**Keywords:** computer-assisted implant surgery, accuracy, pilot drill template, stereolithographic guided template, guided implant surgery

## Abstract

*Background and Objectives*: Implant placement with static navigation enables the reaching of a correct position of implants from an anatomical and prosthetic point of view. Different approaches of static navigation are described in the scientific literature, and the pilot-guided approach is one of the least investigated. The aim of the present study is the evaluation of the accuracy of implant insertion using a pilot drill template. *Materials and Methods*: Fifteen partially edentulous patients, requiring an implant rehabilitation of at least one implant, were enrolled. Pre- and post-operative low-dose CTs were acquired to measure the differences between final positions of implants and virtually planned ones. Three linear discrepancies (coronal, apical, and depth), two angular ones (bucco-lingual and mesio-distal), and the imprecision area were evaluated. Correlations between accuracy and rehabilitated jaws, sectors, and implant length and diameters were also analyzed. *Results:* Forty implants were inserted in fifteen patients using pilot drill templates. Mean coronal deviation was 1.08 mm, mean apical deviation was 1.77 mm, mean depth deviation was −0.48 mm, mean bucco-lingual angular deviation was 4.75°, and mean mesio-distal one was 5.22°. The accuracy was statistically influenced only by the rehabilitated jaw for coronal discrepancy and sectors and implant diameter for bucco-lingual angular deviations. *Conclusions:* The pilot drill template could represent a predictable solution to obtain a correct implant placement. Nonetheless, a safety margin of at least 2 mm should be respected during implant planning to prevent damages to anatomical structures. Therefore, the tool is helpful in order to prosthetically drive the implants; still, great attention must be paid in fully relying on this procedure when approaching dangerous structures such as nerves and vessels.

## 1. Introduction

The use of dental implants for the rehabilitation of partial and total edentulisms is a consolidated procedure and a routine treatment option in the dental professional practice. Numerous studies in the scientific literature report a success rate of more than 95% in all edentulous clinical conditions, evidencing its validity even in the long-term period [1,2,3,4,5].

Computer-guided implant surgery consists of the use of an implant planning software with which, after uploading the patient’s three-dimensional anatomical images acquired from the CT and the prosthetic information obtained from the diagnostic wax-up scans, it is possible to select and virtually place the implant, achieving the best option between the patient’s anatomy and the optimal implant position from an anatomical and prosthetic point of view [6,7].

The pilot drill guide is an hybrid solution for implant placement and is able to combine the advantages of the fully guided and freehand approaches.

It also offers several advantages such as the possibility to reach an accuracy similar to the more precise fully guided template.

The correct positioning of the implant is a key factor for implant success since, if not respected, it leads to an altered biomechanical relationship that could compromise implant survival [8,9,10,11].

One of the options to transfer the virtual planning to the surgery theatre is through a stereolithographic surgical guide, which allows the guidance of osteotomic drills according to the planned position, inclination, and depth, with negligible errors.

The measurement of the entity of these errors is of fundamental interest for the oral surgeon since it makes it possible to know the degree of accuracy of the technique and, consequently, the safety margin to respect during implant planning.

Scientific publications concerning the accuracy of computer-guided implant surgery have mainly investigated the fully guided protocols, whereas the partially guided technique that involves the use of the pilot drill template has been evaluated only in a limited number of studies, i.e., refs. [12,13,14]; furthermore, the latter were conducted with heterogeneous methods, and therefore, their results are difficult to compare for obtaining homogeneous data.

These reasons led to the realization of this study, which aims to investigate the accuracy of implant positioning obtained using the pilot drill template and to evaluate its validity from a clinical point of view.

To limit confounding variables, the present study specifically analyzed implant placement performed by a single expert surgeon with only the pilot drill surgical template, using a single type of implant, and involving exclusively partially edentulous patients, therefore entailing the use of only a dental support template, which, according to the literature, shows the best accuracy.

## 2. Materials and Methods

### 2.1. Study Design

The study was structured as a retrospective survey, including all the implants (40 implants in 15 patients) inserted with a pilot drill template, according to the Nobel Guide™ protocol (Nobel Biocare AB, Göteborg, Sweden), in the period between October 2016 and May 2019, in the Dental and Maxillofacial Clinic, Department of Odontostomatological Surgery, Pediatrics, and Gynecology of the University of Verona.

The study was conducted in accordance with the Helsinki Declaration of 1975, revised in 2013 (ref. [15]), and the protocol was approved by the Local Ethical Committee (1935CESC).

A total of 15 eligible patients were selected and adequately informed about the retrospective nature of the study; once they signed an informed consent drawn up by members board of University of Verona for using their personal and clinical data, they were enrolled in the study.

The inclusion criteria were: patients with partial edentulism (at least 6 healthy supporting template teeth), implant placement using the pilot drill template, use of a single type of implant (NobelActive^®^, Nobel Biocare AB, Göteborg, Sweden), surgeries performed by the same expert oral surgeon, and available post-implant CT (acquired for different clinical reasons).

All the authors applied the NobelGuide^®^ protocol according to the surgical indications.

### 2.2. Pre-Surgical Protocol

After investigating medical and pharmacological anamnesis, the patients were examined to evaluate the possibility of rehabilitating the edentulous area with the computer-guided implant surgery protocol.

The impressions of both arches and the registration of the occlusion were acquired for the realization of the plaster models.

Afterwards, a low-dose CT scan of the dental arch of interest was obtained to evaluate bone volume and quality. The cases with CT images corrupted for scattering were excluded, and the central axis of every implant was used as referring point for measurements. The DICOM (Digital Imaging and Communications in Medicine) data, the standard format for the communication of biomedical data, were subsequently imported into the implant-planning software (DTX Studio Implant™, Nobel Biocare AB, Göteborg, Sweden).

The CT scan was performed by separating the dental arches through the interposition of a radiolucent cotton roller to avoid the possible overlapping between dental elements and consequent erroneous matching with the prosthetic scans. CT scans were performed respecting the parameters for CT recommended by NobelGuide™ protocol.

The dental technician, after obtaining the plaster models, realized the diagnostic wax-up of the missing elements based on the clinician indications, and the models with and without the wax-up were digitized through a laboratory scanner (NobelProcera^®^ 2G System, Nobel Biocare AB, Göteborg, Sweden), saved as “.nxa files”, and sent to the clinician.

In the planning software, through the SmartFusion^®^ (Nobel Biocare AB, Göteborg, Sweden) protocol, the matching between DICOM files and the scans of the dental cast (with and without the wax-up) was performed. The SmartFusion^®^ algorithm ensured precise matching, as it is performed by matching the anatomical information of the dental elements.

The correct position of the fixture was obtained by referring to the anatomical data provided by the CT scan and the prosthetic information obtained from the diagnostic wax-up. The purpose was to select the most appropriate available bone volume for the best implant position within the most suitable prosthetic axis.

The surgical templates were planned without anchor pins considering that, as in the partially edentulous patients, the teeth precisely supported the guide.

After checking the validity of the planning, the implant-prosthetic project was transformed into an acrylic stereolithographic surgical guide.

### 2.3. Surgical Protocol

The correct fitting of the pilot drill template was checked and, if stable and precise, was used to guide the pilot drill.

All surgeries were performed after loco-regional infiltration of anesthesia (mepivacaine hydrochloride 2% and epinephrine 1:100,000). In all cases, a flap was raised up to perform the osteotomic site.

The pilot drill surgical template was housed on the occlusal surface of the teeth, allowing the 2 mm diameter pilot drill to be guided according to the position, angle, and depth of the virtually designed implant; depth stops were used to ensure perfect correspondence between the planning and the actual execution.

After the use of the pilot drill, the surgical template was removed, and the preparation of the implant site was completed freehand using progressively wider diameter drills and following the sequence established by the manufacturer based on the quality of the bone in order to ensure optimal primary stability, especially in case of planned immediate loading.

After completing the preparation of the implant site, the initial insertion of the implant was performed with a low-speed surgical device (25 rpm), while the final insertion was carried out manually.

The used implants were conical and characterized by a double-variable thread self-drilling and self-tapping expanding tapered design with oxidized surface (NobelActive, Nobel Biocare AB, Göteborg, Sweden) and were from 8.5 to 18 mm in length and from 3 to 5 mm in diameter.

At the end of the surgical phase, the following post-operative indications were given to the patient: antibiotic prophylaxis (amoxicillin + clavulanic acid, 1 g every 12 h for 5 days), NSAID therapy, chlorhexidine 0.2% mouthwash (3 times a day), and abstention from physical effort for 1 week.

### 2.4. Superimposition and Variables

The calculation of the three-dimensional deviations between the planned position and the inserted implant were obtained through the superimposition technique, i.e., the overlap of the virtual project with the surgical reality, represented by the post-surgical CT scans.

To achieve this, it was necessary to virtually recreate the surgery by importing it into engineering software.

**Figure 1:**
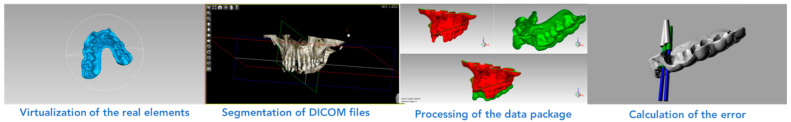
Schematic representation of superimposition’s steps.

The whole process can be divided into four main phases: virtualization of the real elements, segmentation of DICOM files, processing of the data package, and calculation of the error (Figure 1).

For each patient, the plaster models, pilot drill template, and surgical drills were digitized by means a laser scanner (3Shape Wieland D200, 3Shape^®^, Copenhagen, Denmark) with a resolution of 20 microns; then, through the use of the Slicer 3D 4.0 software, the pre and post-operative CT segmentation was carried out in order to obtain the three-dimensional bone volumes.

**Figure 2:**
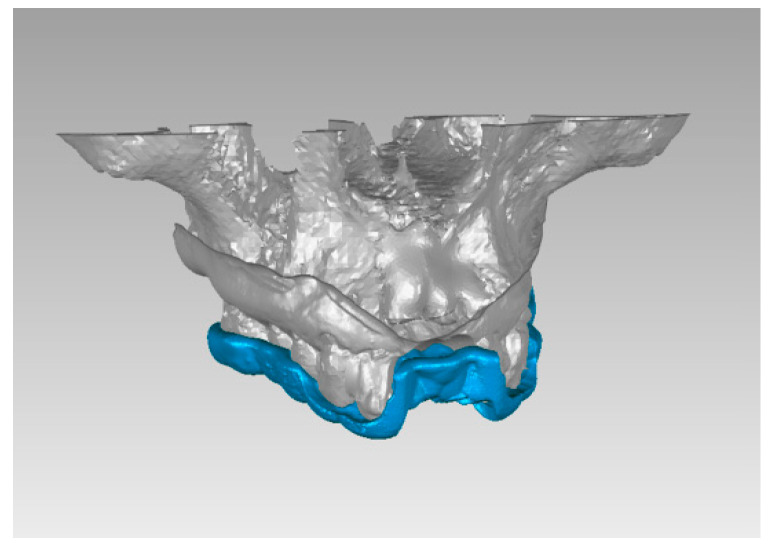
Matching of pre-operative CT, plaster model, and pilot drill template.

The following phase consisted of the realization of the correct spatial relationship between the pre-operative CT, plaster model, and surgical guide (Figure 2) through a dedicated software (Geomagic WRAP 2018 software, Research Triangle Park, NC, USA). With this procedure, a single three-dimensional volume obtained through the use of anatomical reference points was matched to the post-operative bone volume.

**Figure 3:**
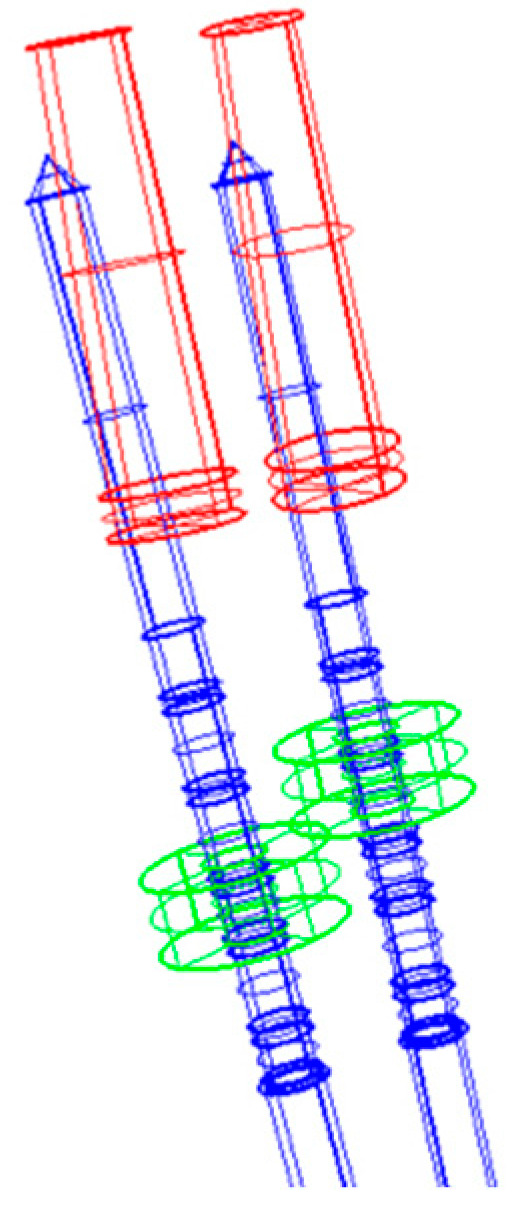
Graphic representation of registered three-dimensional deviations.

The implant volume present in the post-surgical CT was segmented and reconstructed. The same reconstruction procedure was also used for the template sleeves and surgical drills. In this way, it was possible to virtually position the implant drills along the central axis of the sleeves, obtaining the exact position of the planned implants. After placing the real implants in their real positions, three-dimensional deviations could be calculated (Figure 3) using a dedicated software (Rhinoceros^®^ 4.0, McNeel Europe, Barcelona, Spain).

**Figure 4:**
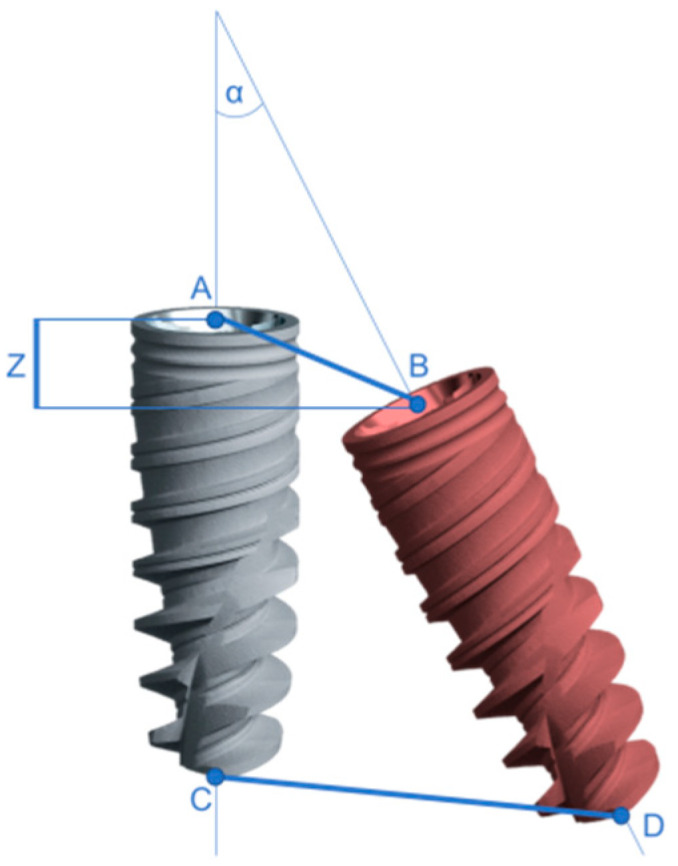
Graphic representation of the linear deviation parameters (coronal, apical, and vertical).

**Figure 5:**
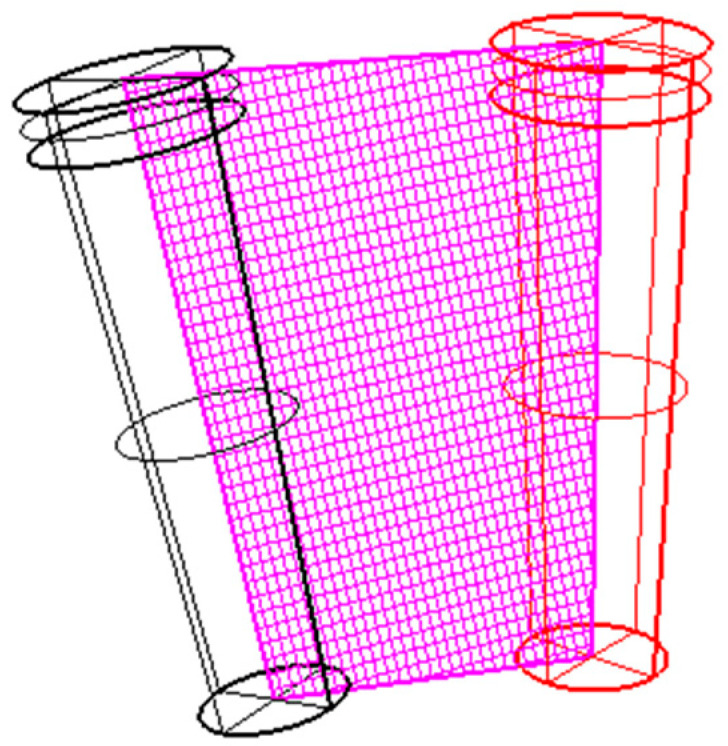
Inaccuracy area calculated between the axes of the implants.

The parameters used in this study to assess the accuracy of implant insertion were the following: total coronal deviation (indicated as distance AB; distance between the coronal center of the planned and the placed implant), total apical deviation (distance CD; distance between the apical center of the planned and placed implant), deviation in depth on the Z axis (distance between the coronal center of the planned implant and a straight line orthogonal to the longitudinal axis of the implant, intersecting the coronal center of the placed implant) (Figure 4), angular deviation calculated both on the bucco-lingual plane (angle made by the axes of the planned and placed implants, measured on the plane transverse to the arch curvature) and mesio-distal plane (angle made by the axes of the planned and placed implants, measured on the plane tangent to the arch curvature), and the area of imprecision (Figure 5).

### 2.5. Statistical Analysis

The obtained measurements were collected, and the created database was imported into the Stata^®^ software (StataCorp LLC, College Station, TX, USA), and a descriptive statistical analysis was performed, obtaining for each measured parameter the average value, the standard deviation, the median, the minimum, and the maximum value.

Then, the non-parametric statistical tests by Wilcoxon–Mann–Whitney, by Kruskal–Wallis, and by Spearman were applied to evaluate the correlation between three-dimensional deviations and the following qualitative variables: rehabilitated dental jaws, rehabilitated arch areas, and implant length and diameter. A *p*-value < 0.05 was considered statistically significant.

## 3. Results

### 3.1. Descriptive Analysis

This study evaluated 40 implants placed in a sample of 15 patients with a mean age of 47.47 years (median 51 years, range 19–66 years). The male sample, consisting of eight patients, represented 20 inserted implants. The same number of implants was inserted in the female sample, consisting of seven patients. The mean number of implants for each patient was 2.67 (range 1–10). Seventeen implants were placed in the maxilla and twenty-three implants in the mandible.

Seventeen implants were inserted in the anterior areas (central and lateral incisor, canine), twelve implants in the middle areas (first and second premolar), and eleven implants in the posterior areas (first and second molar).

In the maxilla, 58.82% of implants were inserted in the anterior areas, 23.53% in the middle areas, and 17.65% in the posterior areas.

In the mandible, 30.43% of the implants was inserted in the anterior areas, 34.78% in the middle areas, and the 34.78% in the posterior areas.

The used NobelActive^®^ implant platforms were 3.0 (10 implants), Narrow Platform (11 implants), and Regular Platform (19 implants).

The mean length of the implants was 11.81 mm (sd ± 2.04 mm, median 11.5 mm): in detail, 5 implants of 8.5 mm (12.5%), 6 implants of 10 mm (15%), 12 implants of 11.5 mm (30%), 13 implants of 13 mm (32.5%), 3 implants of 15 mm (7.5%), and 1 implant of 18 mm (2.5%).

The mean diameter of the implants was 3.84 mm (sd ± 0.68 mm, median 3.5 mm): in detail, 10 implants of 3 mm (25%), 11 implants of 3.5 mm (27.5%), 14 implants of 4.3 mm (35%), and 5 implants of 5 mm (12.5%).

A single type of implant was used to create uniformity in the study.

### 3.2. Accuracy Analysis

The statistical analysis of the accuracy parameters examined, summarized in Table 1, reported the following results:

- Total coronal deviation: mean 1.08 mm, sd ± 0.59 mm, median 1.10 mm, minimum value 0.12 mm, and maximum value 2.60 mm;
- Total apical deviation: mean 1.77 mm, sd ± 0.81 mm, median 1.85 mm, minimum value 0.01 mm, and maximum value 3.18 mm;
- Depth deviation: mean −0.48 mm, sd ± 1.74 mm, median −0.39 mm, minimum value −4.11 mm, and maximum value 2.70 mm;
- Angular deviation on the B-L plane: 4.75° mean, sd ± 2.78°, median 4.69°, minimum value 0°, and maximum value 11.67°;
- Angular deviation on the M-D plane: mean 5.22°, sd ± 3.12°, median 4.78°, minimum value 0°, and maximum value 13.16°;
- Imprecision area: mean 16.96 mm^2^, sd ± 6.32 mm^2^, median 16.37 mm^2^, minimum value 5.69 mm^2^, and maximum value 30.93 mm^2^.

### 3.3. Analysis of Qualitative Variables

(1). Analysis according to the jaw (upper and lower)

In this analysis, the Wilcoxon–Mann–Whitney non-parametric statistical test was applied as the mean values of the variables had to be compared between only two groups.

The mean total coronal deviation was 1.36 mm (sd ± 0.60 mm) for implants inserted in the upper jaw and 0.88 mm (sd ± 0.50 mm) for implants inserted in the lower jaw, and the difference between the two jaws was found to be statistically significant (*p*-value = 0.02).

The mean total apical deviation was 1.99 mm (sd ± 0.85 mm) for implants inserted in the upper jaw and 1.61 mm (sd ± 0.75 mm) for implants inserted in the lower jaw, and the difference between the two jaws was found not to be statistically significant (*p*-value = 0.12).

The mean depth deviation was −0.73 mm (sd ± 2.27 mm) for implants inserted in the upper jaw and −0.30 mm (sd ± 1.23 mm) for implants inserted in the lower jaw, and the difference between the two jaws was found not to be statistically significant (*p*-value = 0.57).

The angular deviation on the mean B-L plane was 4.85° (sd ± 2.80°) for implants inserted in the upper jaw and 4.68° (sd ± 2.83°) for implants inserted in the lower jaw, and the difference between the two jaws was found not to be statistically significant (*p*-value = 0.63).

The angular deviation on the mean M-D plane was 4.54° (sd ± 2.89°) for implants inserted in the upper jaw and 5.73° (sd ± 3.25°) for implants inserted in the lower jaw, and the difference between the two jaws was found not to be statistically significant (*p*-value = 0.16).

(2). Analysis according to the arch areas (anterior, medium, and posterior)

In this analysis, it was necessary to compare the mean values of the ordinal variables among more than two groups; therefore, the Kruskal–Wallis non-parametric statistical test was applied.

The mean total coronal deviation was 0.95 mm (sd ± 0.41 mm) for implants inserted in the anterior areas, 1.13 mm (sd ± 0.66 mm) in the middle areas, and 1.25 mm (sd ± 0.74 mm) in the posterior areas, and the difference was not statistically significant (*p*-value = 0.56).

The mean total apical deviation was 1.72 mm (sd ± 0.73 mm) for implants inserted in the anterior areas, 1.75 mm (sd ± 0.79 mm) in the middle areas, and 1.88 mm (sd ± 0.99 mm) in the posterior areas, and the difference was not statistically significant (*p*-value = 0.72).

The mean depth deviation was −1.18 mm (sd ± 1.81 mm) for implants inserted in the anterior areas, 0.17 mm (sd ± 1.23 mm) in the middle areas, and −0.12 mm (sd ± 1.84 mm) in the posterior areas, and the difference was not statistically significant (*p*-value = 0.14).

The mean angular deviation on the B-L plane was 3.33° (sd ± 2.30°) for implants inserted in the anterior areas, 6.42° (sd ± 2.61°) in the middle areas, and 5.13° (sd ± 2.71°) in the posterior areas, and the difference of areas was statistically significant (*p*-value = 0.02).

The mean angular deviation on the M-D plane was 5.60° (sd ± 3.67°) for implants inserted at the level of the anterior areas, 4.12° (sd ± 2.69°) in the middle areas, and 5.85° (sd ± 2.52°) in the posterior areas, and the difference of areas was not statistically significant (*p*-value = 0.31).

(3). Analysis according to the length of the implant

To assess the existence of a correlation between implant length and each single measured parameter, following an increasing or decreasing trend, the Spearman non-parametric statistical test was applied.

Based on the results obtained from this test performed for each parameter, it was possible to conclude that the values of the total coronal deviation, total apical deviation, depth deviation, angular deviation on the B-L plane, and angular deviation on the M-D plane were not significantly statistically correlated with the implant length due to the fact that the *p*-values obtained were, respectively, *p*-value = 0.17, *p*-value = 0.85, *p*-value = 0.24, *p*-value = 0.95, and *p*-value = 0.20.

(4). Analysis according to the implant diameter

To assess the existence of a correlation between implant diameter and each single measured parameter, following an increasing or decreasing trend, the Spearman non-parametric statistical test was applied.

Considering the results obtained from this tests performed for each parameter, it was possible to assess that the values of the total coronal deviation, total apical deviation, depth deviation, and angular deviation on the M-D plane were not significantly statistically correlated with the implant diameter variable due to the fact that the *p*-values obtained were, respectively, *p*-value = 0.05, *p*-value = 0.23, *p*-value = 0.58, and *p*-value = 0.32. Otherwise, the correlation between angular deviation on the B-L plane and diameter implant was found to be statistically significant (*p*-value = 0.004).

## 4. Discussion

The correct three-dimensional position of the implants is a key factor for achieving an optimal aesthetic and long-term outcome of prosthetic rehabilitations [16]. A recent study investigated the risk of peri-implantitis in a sample of 332 implants, concluding that almost half of the cases were related to an inadequate implant positioning [17].

Computer-guided surgery allows the clinician to place the implants in their ideal position, both from the anatomical and prosthetic point of view, reducing the risk of possible complications [18].

All patients involved in the study had partial edentulism, and consequently, all the surgical templates had a dental support condition ensuring the best stability and precision.

In the interpretation of these results, it should be considered that some values represent a deviation from the planning that occurred after the use of the guided pilot drill, made necessary by the clinical conditions found intraoperatively. For example, an implant inserted deeper than the planned position may be due to the need to obtain an adequate torque for immediate loading. In general, the means of the deviations measured in this study are included within a 2 mm safety perimeter from the planned implant profile. This perimeter defines the safety area that must always be considered during computer-guided implant planning [19,20,21,22,23].

The greater precision reported for the lower jaw, i.e., refs. [24,25], can be explained considering that the dense mandibular bone is able to limit the oscillations of the drills.

On the other hand, the greater precision of the implants inserted in the anterior areas is probably related to the easier visibility and space availability [26]. Otherwise, the greater deviations found in the posterior areas could be explained with a difficult access, the optical distortion of the lateral vision, and a lower bone density, mainly in the upper jaw.

It is possible to observe a gradual increase in the total apical deviation for 10, 11.5, 13, and 15 mm length implants, probably related to the fact that since there is a tolerance between the drill and the template sleeve, with the same angular deviation, longer implants exhibit greater apical deviations [27,28]. Furthermore, a study by Lal et al. reported that a tolerance of 0.2–0.3 mm can produce an angular deviation greater than 5° [29]. However, these differences, which are statistically not significant, may represent system tolerance.

Implant diameter analysis showed that angular deviation on the B-L plane increases with statistical significance (*p*-value = 0.004) in relation to the implant diameter; for the other parameters, the correlation with the implant diameter was not statistically significant.

**Figure 6:**
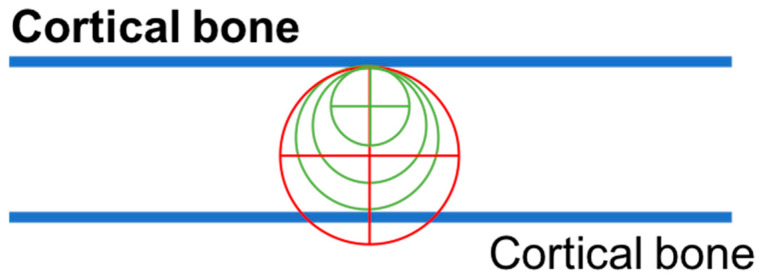
Explanatory drawing of the greatest deviation on the B-L plane with wider drills.

The greatest deviation on the B-L plane is interpretable considering that wider drills coming in contact with the dense cortical bone could be deviated in the direction of least resistance areas (Figure 6).

The limited number of cases, dictated by the restricted inclusion criteria, made possible the study of a homogeneous sample of computer-guided surgical cases containing only patients who presented partial edentulism and were treated by the same operator using the same implant and the same surgical template; this could explain why some statistic tests were not statistically significant.

The only few previous publications investigating the use of pilot drill templates, i.e., 11–14, even if conducted in different conditions, are summarized in Table 2.

The most suitable study for a direct comparison is an RCT published by Younes in 2018 [14], including only partially edentulous cases and using similar methods and comparing the results obtained with freehand, pilot drill, and totally guided surgeries.

Only another RCT by Vercruyssen investigated the accuracy of the pilot drill but was conducted with different inclusion criteria [12]; the authors did not use a pilot drill surgical guide obtained through a stereolithographic digital process but an analogical pilot drill template prepared by the dental technician, treating complete edentulous subjects using a less accurate bone- or mucous-supported template.

Finally, comparing our results with data reported in a recent systematic review by Tahmaseb [30], investigating the accuracy of fully template, it is possible to state that the pilot drill mode allows an accuracy similar to the more precise fully guided template; in fact, considering only partially edentulous patients, fully guided and pilot drill templates showed similar outcomes.

The pilot drill surgical guide represents a hybrid solution for implant placement, which is able to combine the advantages of computer-guided surgery with those of conventional technique; virtual planning allows to evaluate and prevent in advance possible complications, reducing the operator’s stress. To achieve a high level of precision, the drills following the pilot one must be passively inserted in order to minimize deviations. The pilot drill template shows several benefits if compared to a fully guided one: greater freedom of treatment and visibility, possibility to perform osteoplasty or regenerative procedures, real torque perception, lower cost, easy use, less operator-dependent intervention, and reduction of overheating [31,32,33,34,35].

A limitation of the present study is the low number of involved patients, which is related to strict inclusion criteria, and future clinical trials with a greater number of cases are planned to confirm the results evidenced in this study. A further limitation is represented by the use of radiological images (already available for different clinical purposes) instead an intraoral scanner, which was not available during this clinical trial.

All the authors are aware that further strategies will involve dynamic guide surgery, but the learning curve could be too challenging.

## 5. Conclusions

The data that emerged from this study confirmed that the pilot drill technique allows, in partially edentulous patients, a satisfactory level of accuracy.

The pilot drill protocol, a hybrid solution between a fully guided and freehand approach, makes it possible to combine their benefits, limiting the disadvantageous aspects. The mean three-dimensional deviations evaluated in this study confirmed the importance of maintaining, during implant planning, a safety margin of at least 2 mm from the relevant anatomical structures.

The pilot-drill-guided surgery is therefore proposed as a technique to be used in situations where greater accuracy is required, such as atrophic and aesthetic cases, but when, due to the wider size of the sleeves, it is not possible to use the fully guided template (e.g., very narrow interdental spaces); a further but not negligible advantage of pilot drill use is a reduction of production costs.

## Figures and Tables

**Table 1:** Summarized statistical analysis of the examined accuracy parameters.

	Mean (±Standard Deviation)	Median (Min-Max Value)	*p*-Value
Total coronal deviation	1.08 (0.59)	1.10 (0.12–2.60)	<0.001
Total apical deviation	1.77 (0.81)	1.85 (0.01–3.18)	<0.001
Depth deviation	−0.48 (1.74)	−0.39 (−4.11–2.70)	0.087
Angular deviation on B-L plane	4.75 (2.78)	4.69 (0–11.67)	<0.001
Angular deviation on M-D plane	5.22 (3.12)	4.78 (0–13.16)	<0.001
Imprecision area	16.96 (6.32)	16.37 (5.69–30.93)	<0.001

**Table 2:** Comparison of the results of the present study with previous publications investigating the use of pilot drill template.

Author	Total Coronal Deviation	Total Apical Deviation	Depth Deviation	Angular Deviation on B-L Plane	Angular Deviation on M-D Plane
Vercruyssen et al., 2014 [12,13]	2.97 ± 1.41 mm	3.40 ± 1.68 mm	2.20 ± 1.44 mm	8.43 ± 5.10°	
Younes et al., 2018 [14]	1.12 ± 0.49 mmd	1.43 ± 0.88 mm	0.68 ± 0.44 mm	5.95 ± 4.26°	
Tahmaseb et al., 2018 [30]	0.9 mm (CI 95%: 0.79–1.00)	1.2 mm (CI 95%: 1.11–1.20)	0.2 mm (CI 95%: 0.25–0.57)	3.3°(CI 95%: 20.7–4.63)	
Present study	1.08 ± 0.59 mm	1.77 ± 0.81 mm	−0.48 ± 1.74 mm	4.75 ± 2.78°	5.22 ± 3.12°

## Data Availability

Not applicable.
